# Self-collection of capillary blood using Tasso-SST devices for Anti-SARS-CoV-2 IgG antibody testing

**DOI:** 10.1371/journal.pone.0255841

**Published:** 2021-09-02

**Authors:** Tess Hendelman, Anu Chaudhary, Angela C. LeClair, Kimberly van Leuven, Jacqueline Chee, Susan L. Fink, Emily J. Welch, Erwin Berthier, Brook A. Quist, Anna Wald, Mark H. Wener, Andrew N. Hoofnagle, Chihiro Morishima

**Affiliations:** 1 Department of Laboratory Medicine and Pathology, University of Washington, Seattle, WA, United States of America; 2 Department of Medicine, University of Washington, Seattle, WA, United States of America; 3 Tasso, Inc., Seattle, WA, United States of America; 4 Department of Epidemiology, University of Washington, Seattle, WA, United States of America; 5 Vaccine and Infectious Diseases Division, Fred Hutchinson Cancer Research Center, Seattle, WA, United States of America; University of Nevada Reno School of Medicine, UNITED STATES

## Abstract

**Background:**

Efforts to minimize COVID-19 exposure during the current SARS-CoV-2 pandemic have led to limitations in access to medical care and testing. The Tasso-SST kit includes all of the components necessary for remote, capillary blood self-collection. In this study, we sought to investigate the accuracy and reliability of the Tasso-SST device as a self-collection device for measurement of SARS-CoV-2 IgG antibodies.

**Methods:**

Capillary blood was obtained via unsupervised and supervised application of the Tasso-SST device, and venous blood was collected by standard venipuncture. Unsupervised self-collected blood samples underwent either extreme summer or winter-simulated shipping conditions prior to testing. Sera obtained by all three methods were tested concurrently using the EuroImmun anti-SARS-CoV-2 S1 IgG assay in a CLIA-certified clinical laboratory.

**Results:**

Successful Tasso-SST capillary blood collection by unsupervised and supervised administration was completed by 93.4% and 94.5% of participants, respectively. Sera from 56 participants, 55 with documented (PCR+) COVID-19, and 33 healthy controls were then tested for anti-SARS-CoV-2 IgG antibodies. Compared to venous blood results, Tasso-SST-collected (unstressed) and the summer- and winter-stressed blood samples demonstrated Deming regression slopes of 1.00 (95% CI: 0.99–1.02), 1.00 (95% CI: 0.98–1.01), and 0.99 (95% CI: 0.97–1.01), respectively, with an overall accuracy of 98.9%.

**Conclusions:**

Capillary blood self-collection using the Tasso-SST device had a high success rate. Moreover, excellent concordance was found for anti-SARS-CoV-2 IgG results between Tasso-SST capillary and standard venous blood-derived sera. The Tasso-SST device should enable widespread collection of capillary blood for testing without medical supervision, facilitating epidemiologic studies.

## Introduction

The global SARS-CoV-2 pandemic has forced large numbers of people into various degrees of physical isolation and quarantine, increasing barriers to health care, laboratory testing, and epidemiological surveillance [1]. In this context, we sought to investigate the feasibility and performance of an in-home capillary blood collection device, Tasso-SST, for anti-SARS-CoV-2 IgG serological testing at a CLIA-certified clinical laboratory.

The Tasso-SST kit contains all the necessary components for an in-home capillary blood draw, including instructions, shipping materials, and a self-use blood collection device. The device includes an adhesive strip for attaching the device to the upper arm, a lancet assembly triggered by a large red button, and a collection tube with a separator gel designed to collect up to approximately 600 μL of capillary blood (https://www.tassoinc.com). Among other applications, the Tasso device has previously been employed to measure drug levels in patients treated with an anti-A therapeutic antibody [2].

We describe our single-center experience using the Tasso-SST capillary blood collection device among volunteers and persons recovered from virologically documented COVID-19. We evaluated the success of obtaining blood for testing using the Tasso-SST device, either supervised or unsupervised. We then compared antibody levels detected in capillary blood obtained using the Tasso-SST device with venous blood obtained by standard venipuncture. Finally, capillary blood obtained via the Tasso-SST device was subjected to extreme temperature conditions that could potentially occur with shipping to evaluate the effects on antibody test results.

## Materials and methods

### Patient enrollment

Male and female participants, ages 21 through 73, underwent University of Washington IRB-approved (#4312) written informed consent and study procedures at the University of Washington Virology Research Clinic in Seattle, WA between June and August 2020. Participants with documented evidence (serologic or RT-PCR) of prior SARS-CoV-2 infection who had been recruited as potential therapeutic plasma donors were enrolled in the current study; subjects must have been asymptomatic, afebrile for 14 or more days, be 28 days past the resolution of their COVID-19 illness, and were eligible to become a donor if their anti-SARS-CoV-2 neutralizing antibody titer was at least 1:80. Subjects must have met FDA-approved criteria per local blood collector for plasmapheresis, and be judged to have adequate peripheral venous access for plasmapheresis donation. Healthy controls included male and female participants, ages 25 through 69, recruited between June and August 2020. These participants were required to have no known history of COVID–19 illness. Green’s rule of thumb calculation for a linear regression (m = 1) for medium effect size yielded a sample size of 58 [3]. The sample size was then increased to allow for potential samples with inadequate collection for comparing ELISA results.

### Sample collection, handling and processing

**Fig 1:**
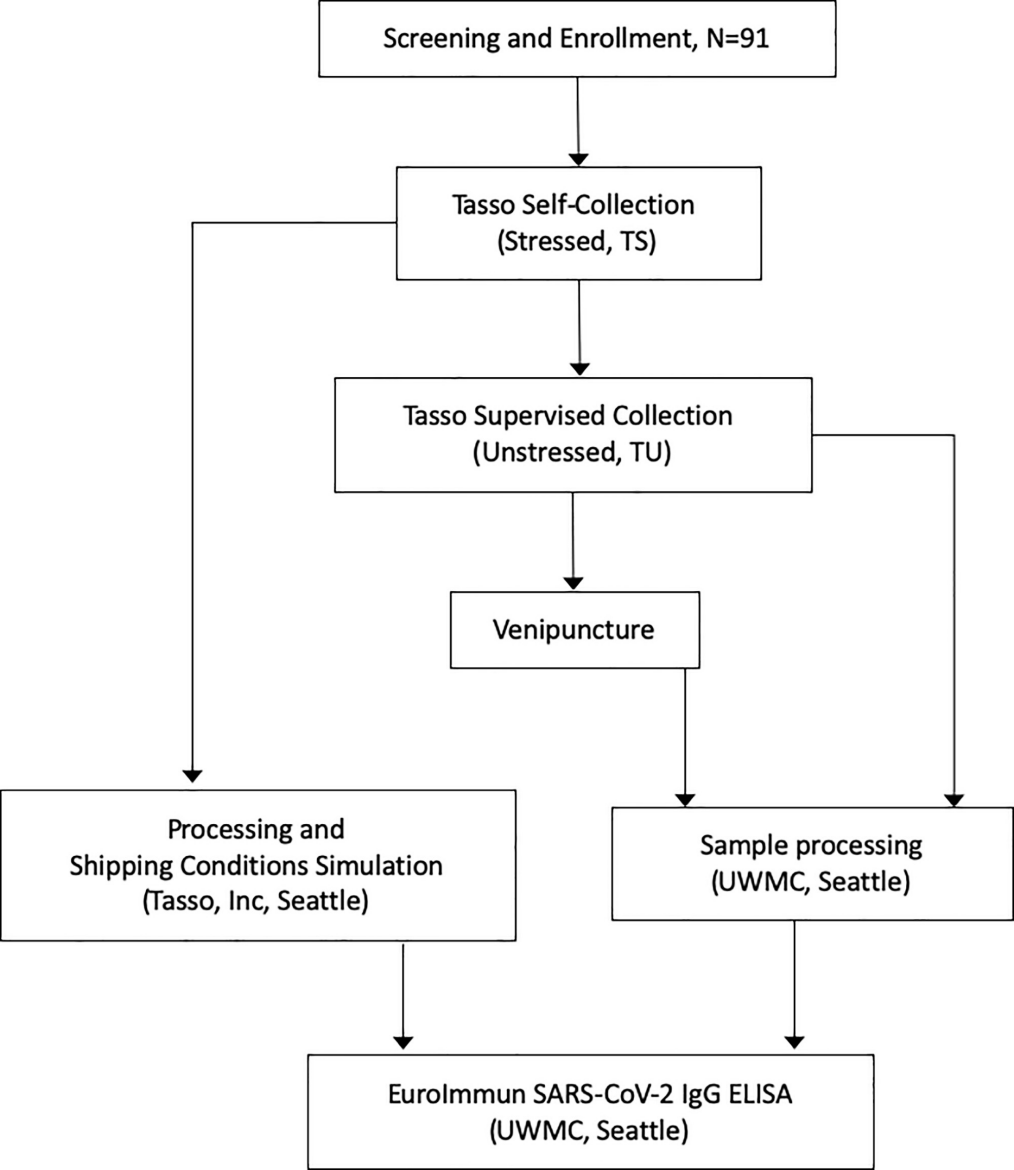
Study schema to determine the usability of the Tasso device for self-collection of capillary blood. Two of 91 subjects were excluded due to inability to obtain blood samples; 56 convalescent and 33 healthy subjects had blood samples tested.

Capillary blood specimens were collected with the Tasso-SST blood sampling kit (Tasso, Inc., Seattle, WA). The study design and flow of samples is outlined in Fig 1. Using the provided kit instructions and kit contents, participants collected the first Tasso-SST blood sample (Tasso-stressed sample) in a self-administered, unsupervised manner. A health care professional collected the second Tasso-SST sample (Tasso-unstressed sample). Lastly, a serum venous sample was collected in a BD SST™ (gold top) tube (BD, Franklin Lakes, NJ). Tasso-stressed samples were sent at ambient temperature from the clinic to Tasso, Inc. (Seattle, WA) and Tasso-unstressed sample and venous SST tubes were sent to the University of Washington Medical Center (UWMC) Clinical Immunology Laboratory. On arrival, unstressed Tasso-SST tubes and venous SST tubes were centrifuged for 10 minutes at 3254xg at 25°C and then stored at 4°C until all Tasso-unstressed, Tasso-stressed and venous samples from the same volunteer were available to test on the same run, typically within 1 week of collection. To simulate delays between collection and shipping, self-collected Tasso-stressed samples were stored at uncontrolled room temperature for 8 to 19 hours. To simulate summer (N = 45) or winter (N = 44) shipping, samples were then subjected to a modified ISTA 7D 2007 shipping standard (48-hour domestic freight transport) at Tasso, Inc. The summer protocol included the following cycles: 8 hr at 40°C, 4 hr at 22°C, 2 hr at 40°C, 36 hr at 30°C, then 6 hr at 40°C. The winter protocol included the following cycles: 8 hr at -10°C, 4 hr at 18°C, 2 hr at -10°C, 36 hr at 10°C, then 6 hr at -10°C. Samples were centrifuged as outlined above, then transported to the UWMC Clinical Immunology Laboratory for testing. The mean number of hours from specimen collection to processing following simulated shipping was 72.7 (median 73.0, interquartile range 70.6–74.6, range 62.5–84.3). Tasso collection tubes were weighed, and weights converted using the formula: total tube weight in grams = (blood volume in mLs x 1.066) + 1.78.

### Serologic testing

Serologic testing was performed using an FDA-authorized (EUA), CE-marked EuroImmun (Germany) anti-SARS-CoV-2 IgG kit [4]. The EuroImmun (EU) manual immunoassay measures antibodies to recombinant structural protein (S1 domain) yielding quantitative values, and is authorized to provide qualitative results. All testing and analyses were performed according to the manufacturer’s protocols using 10 microliters of patient serum per test, with the OD ratio calculated using the kit calibrator; the manufacturer-provided reference range is as follows: ratio <0.8 (negative), ratio ≥ 0.8 to <1.1 (borderline), and ratio ≥ 1.1 (positive).

### Statistical analyses

Statistical analyses were performed using GraphPad Prism software version 9.0.0 (San Diego, CA) and R version 3.4.4 [5].

## Results

### Study participants

The characteristics of the 89 participants whose samples underwent Anti-SARS-CoV-2 IgG testing are outlined in Table 1. Of these 89 participants, 56 had recovered from COVID-19, and 33 asymptomatic controls had no history of exposure to SARS-CoV-2. This cohort was generally well-educated, with 85/89 (95.5%) having some college education or beyond, and 4/89 (4.5%) having attended technical or trade school.

**Table 1:** Study participant characteristics, N = 89.

Characteristic	Numbers
Median Age (Range)	45 (21–73)
Male Gender (%)	30 (33.7)
White (%)	68 (76.4)
Asian (%)	11 (12.4)
Black (%)	5 (5.6)
Hispanic (%)	7 (7.9)
Any college education & above (%)	85 (95.5)
Technical/trade school (%)	4 (4.5%)
Median Days Since PCR+ (Range)^*a*^	96 (36–128)

^*a*^Median Days Since PCR+ excludes 1 participant whose date of PCR-positivity was not available, N = 55; 33 participants were controls.

### Successful blood collection using the Tasso device

Sample collection was attempted from 91 participants enrolled in the study (Table 2). Of these 91 participants, 2 were excluded from antibody result evaluation due to unsuccessful blood collection: one was excluded after multiple unsuccessful phlebotomy attempts and one after two unsuccessful attempts of a provider administering the Tasso-SST device. No participants were unsuccessful for Tasso-SST self-collection. Eight participants self-collected capillary blood twice via the Tasso-SST device as the health care professional deemed the first sample to be low volume. For 2 of these 8 subjects, the second sample was disregarded as analysis was performed successfully on the first sample; thus only 6 participants “required” a second Tasso self-collection to produce an adequate specimen for testing, as shown in Table 2. A provider-administered second Tasso collection was required for 5 participants in order to obtain sufficient blood volumes for testing, and one of these was considered “unsuccessful”. For the initial participant self-collected samples, the mean volume collected was 339 μL (inter-quartile range: 228–454 μL); similar volumes were obtained with supervised collection (mean 311 μL, inter-quartile range: 186–423 μL). For additional reference, assuming an average hematocrit of 42% (average for both genders together), we estimate that 78 of 89 samples (87.6%) would have yielded 100 microliters of serum, and 64/89 (71.9%) would have yielded 150 microliters of serum. No patients required redraws for both self-collection and administered collection using the Tasso-SST device.

**Table 2:** Sample collection for 91 participants.

Blood Draw Method	Successful^*a*^ ^–^First Draw, N (%)	Redraw Required^*b*^, N (%)	Unsuccessful^*c*^ Blood Draw, N
Venous Draw	90 (98.9)	1 (1.1)	1
Tasso–Administered Draw	86 (94.5)	5 (5.5)	1
Tasso–Self Draw	85 (93.4)	6 (6.6)	0

^*a*^Successful defined as sample and test result obtained.

^*b*^Redraw required to obtain test result; includes unsuccessful draws.

^*c*^Unsuccessful defined as no sample collected for testing; testing not done.

### Correlation of anti-SARS-CoV-2 IgG results between venous and capillary (Tasso) blood

The distribution of anti-SARS-CoV-2 IgG results according to the method of sample collection and handling is shown in Table 3. Using the manufacturer-provided cutoffs, only 1 venous sample (BD gold top SST tube collection) was in the borderline range, while 40 were negative and 48 were positive. The one sample with borderline venous results was found to be negative in both the Tasso-unstressed and Tasso-stressed samples, although the value of 0.802 in the venous sample (BD gold top SST tube) just met the borderline threshold of > = 0.8. Serum drawn and tested concurrently from a BD red top serum tube exhibited an OD ratio of 0.758 and was considered negative. These quantitative differences were within the expected variability of EuroImmun assay results and obtained ~4.5 months after first COVID-19 symptoms. The 88 remaining Tasso results fell in the same positive and negative categories as the venous results, for an overall resulting accuracy of 98.9%.

**Table 3:** Distribution of anti-SARS-CoV-2 IgG results among study participants according to blood draw conditions (N = 89).

Interpretation	EuroImmun Thresholds	Venous Blood, N	Tasso-Unstressed, N	Tasso- Stressed^a^, N
Negative	<0.8	40	41	41
Borderline	≥0.8<1.1	1	0	0
Positive	≥1.1	48	48	48

^a^Tasso-Stressed includes summer and winter conditions combined; the one discordant sample was winter-stressed

**Fig 2:**
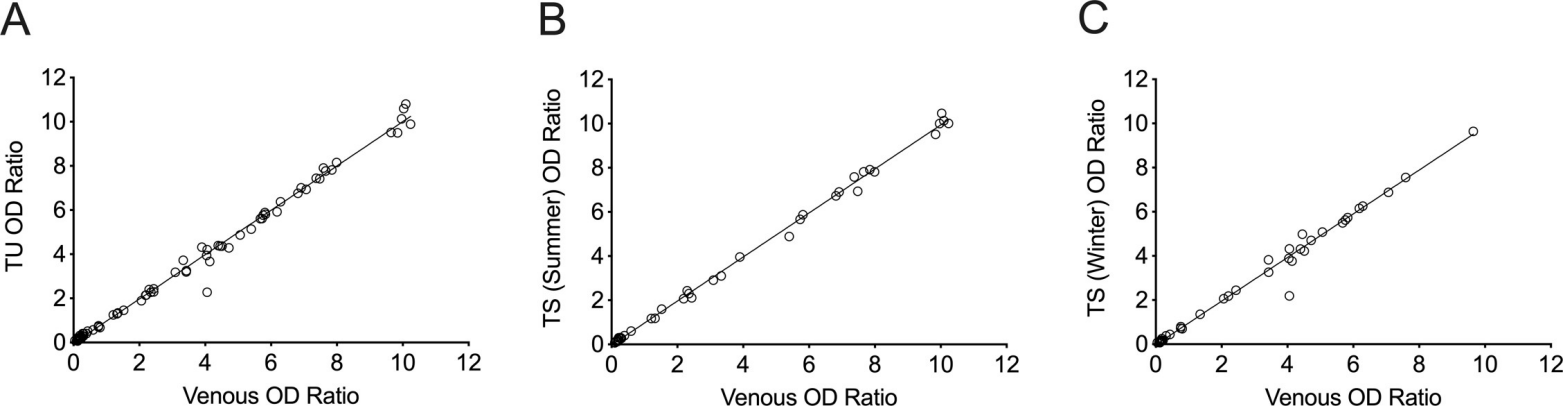
EuroImmun anti-SARS-CoV-2 IgG results obtained from standard venous phlebotomy and capillary blood collected using Tasso-SST devices. Each circle represents results from an individual participant. Correlation between EuroImmun OD ratios from venous blood and provider-administered Tasso device collected capillary blood (Tasso Unstressed (TU), N = 89, p<0.0001, Deming regression: Y = 1.00*X—0.04) (A). Correlation between venous samples results and those from stressed Tasso-SST (TS) samples under summer (B, N = 44, p<0.0001, Deming regression: Y = 1.00 *X– 0.03) or winter conditions (C, N = 45, p<0.0001, Deming regression: Y = 0.99*X– 0.03).

The pair-wise correlation between the OD ratios obtained after blood collection by standard venipuncture and Tasso-SST devices is shown in Fig 2. It is noteworthy that all three blood samples for each participant were tested in the same run, to eliminate any effect of inter-assay variation. Results obtained from venous blood and the unstressed Tasso-SST device demonstrated slopes for the Deming and linear regression analyses of 1.00 with 95% confidence intervals (CI) of 0.99–1.02 and 0.98–1.02, respectively.

The self-collected Tasso-SST samples underwent a 48-hour shipping simulation as described in Materials and Methods. Anti-SARS-CoV-2 IgG results from both summer- (N = 44) and winter-stressed samples (N = 45) were compared to venous samples that underwent routine processing procedures after collection (Fig 2B, 2C). A comparison of EuroImmun results obtained from summer-stressed Tasso-SST and venous samples indicated slopes for Deming and linear regression of 1.00 (95% CI: 0.98–1.01). Similarly, a comparison of EuroImmun results obtained from winter-stressed Tasso-SST and venous samples indicated slopes for Deming and linear regression of 0.99 (95% CI: 0.97–1.01) and 0.98 (95% CI: 0.95–1.00), respectively.

## Discussion

We investigated the performance of a capillary blood collection device intended for self-application and shipping to a clinical laboratory for subsequent anti-SARS-CoV-2 S1 IgG antibody testing. Our findings indicated that the Tasso-SST device was used successfully to collect adequate volume for testing by a manual ELISA 93.4% of the time, compared to 98.9% for venous blood draw by a trained professional. Moreover, we showed that quantitative antibody levels detected in the venous and Tasso-SST capillary serum were highly correlated, even when the samples were stressed by winter and summer conditions (to mimic extreme shipping conditions) compared to clinic-obtained venous blood samples that were promptly delivered to the laboratory.

The window of time between PCR-positivity and anti-Spike IgG testing in our study was relatively narrow at 36–128 days. However, the EuroImmun anti-Spike assay results from blood collections during this interval encompassed a relatively broad range of OD ratio values (0.05 to 10.8 OD ratios) for the 89 enrolled subjects. While higher antibody levels may be present earlier after infection among hospitalized patients compared to the outpatients we tested, it is unlikely that the Tasso device would be utilized in that population. The most relevant concern would be the correlation between antibody levels in venous and capillary blood at lower levels; half of the samples we tested yielded results between 0.2 and 5.4, which encompasses both low-positive and negative values. The excellent correlation between results from venous and capillary blood provide confidence that the material type collected via Tasso will allow for the accurate measurement of low antibody levels using high quality EUA SARS-CoV-2 ELISAs.

Unsupervised self-collection of blood samples at home has several potential limitations. One potential limitation is the need for adequate education of patients so that the device is correctly applied and samples shipped to the laboratory. In our study, 94.9% of participants successfully self-collected capillary blood; the high educational attainment of our cohort may have contributed to the high success rate. Outside of the current study, Tasso, Inc. evaluated an additional 20 individuals who had no prior experience with sample self-collection; 13 had attended “some college”, 3 had attended a trade or technical school, and 3 were high school graduates. Among these 20 subjects, 17 (85%) were able to successfully self-collect an acceptable sample on their first attempt following the kit instructions for performing the collection.

Other potential limitations include the time needed for shipping prior to specimen processing (resulting in a delay of testing) and potential adverse environmental (or climatic) conditions associated with shipping that could affect the stability of the analyte to be tested. However, as shown here, antibodies tend to be stable analytes; the mean elapsed time between Tasso sample collection and processing was 72.7 hours, a relatively long period of time. Hodgkinson and colleagues also evaluated the effect of delayed processing and temperature on results from a multiplex immunoassay measuring antibodies against various infectious antigens and found their results to be stable at room temperature through 6 days [6]. Their results are consistent with our clinical experience evaluating the time-dependent stability of samples tested by antibody binding assays.

A final limitation of this self-collection approach is the volume of blood that can be obtained using the Tasso-SST device. In the current study, the average whole blood volume obtained by Tasso-SST self-collection was 339 μL, which corresponds to approximately 196 μL of serum. Consequently, 93.4% of samples had sufficient volume for testing by our manual ELISA, which required only 10 μL. While this small sample volume is typical of manual ELISAs, the overall volume limitation could be an issue for analytes measured using automated instruments, since they typically require larger serum volumes for testing.

An alternative approach to remote blood collection for subsequent laboratory testing is the use of dried blood spots (DBS) [7–9], performed by having the patient squeeze a small amount of capillary blood onto paper cards after fingerprick. The advantage of this method of blood collection is that it is straightforward to perform, is stable during shipping, is inexpensive, and has been used for decades to test other analytes, primarily for neonatal screening of diseases such as phenylketonuria and congenital hypothyroidism. In fact, Perkin Elmer recently released a CE-marked dried blood spot-based anti-SARS-CoV-2 IgG assay on its GSP®/DELFIA® platform [10]. However, the collection procedure can be more painful because of the pain sensitivity of the anatomic site (fingertip vs. upper arm), smaller volumes are collected, and additional steps are needed at the laboratory to extract the analytes from the paper cards. The sensitivity of the testing may be diminished as a consequence. It is currently unclear how the DBS collection method compares to the Tasso-SST device for generating anti-SARS-CoV-2 antibody results. While DBS may allow for longer analyte stability prior to testing, the requirement for additional processing steps prior to analysis and potential for decreased assay sensitivity are all factors that will need to be considered for particular testing scenarios.

The current global pandemic has led to restricted movement and gathering of people to mitigate the spread of viral infection. In this setting, telemedicine has emerged as an increasingly common alternative to in-person medical visits [11]; access to a self-collection device for blood monitoring may be a useful adjunct. An in-home self-collection device such as Tasso-SST could also facilitate wide-spread immunosurveillance studies of SARS-CoV-2 infection. Moreover, many other analytes, including other antibodies and small molecules, could be assayed using blood samples collected with this device. Our data support the utility of Tasso-SST for blood collection demonstrating equivalence of results obtained from standard venous and self-collected capillary blood for measurement of anti-SARS-CoV-2 S1 IgG antibodies. These findings pave the way for exploring the use of this device for measuring other antibodies and analytes, particularly when access to appropriate testing is limited by disability, distance, travel barriers, safety concerns, or other factors.

## Supporting information

S1 Dataset Raw data file for comparison of Tasso-SST capillary blood to serum for SARS-CoV-2 anti-Spike IgG antibody testing. (XLSX)
